# Correction: A hybrid blue perovskite@metal–organic gel (MOG) nanocomposite: simultaneous improvement of luminescence and stability

**DOI:** 10.1039/d2sc90032g

**Published:** 2022-03-02

**Authors:** Samraj Mollick, Tarak Nath Mandal, Atanu Jana, Sahel Fajal, Sujit K. Ghosh

**Affiliations:** Department of Chemistry, Indian Institute of Science Education and Research (IISER) Pune Dr. HomiBhabha Road, Pashan Pune 411008 India sghosh@iiserpune.ac.in; Center for Superfunctional Materials, Department of Chemistry, School of Natural Science, Ulsan National Institute of Science and Technology (UNIST) Ulsan 44919 South Korea; Centre for Energy Science, IISER Pune Pune 411008 India

## Abstract

Correction for ‘A hybrid blue perovskite@metal–organic gel (MOG) nanocomposite: simultaneous improvement of luminescence and stability’ by Samraj Mollick *et al.*, *Chem. Sci.*, 2019, **10**, 10524–10530, DOI: 10.1039/C9SC03829A.

The authors regret that the formula used for the 2D perovskite EAPbBr_3_ throughout the text is incorrect. The formula should read EA_2_PbBr_4_.

The Royal Society of Chemistry apologises for these errors and any consequent inconvenience to authors and readers.

## Supplementary Material

